# Factors Influencing the Introduction of Value-Based Payment in Integrated Stroke Care: *Evidence from a Qualitative Case Study*

**DOI:** 10.5334/ijic.7566

**Published:** 2023-08-10

**Authors:** Newel Salet, Bianca I. Buijck, Dianne H. K. van Dam-Nolen, Jan A. Hazelzet, Diederik W. J. Dippel, Erik Grauwmeijer, F. T. Schut, Bob Roozenbeek, Frank Eijkenaar

**Affiliations:** 1 Erasmus School of Health Policy & Management, Erasmus University, NL; 2 Rotterdam Stroke Service, The Netherlands; 3 Erasmus MC University Medical Center, Department of Neurology, Rotterdam, NL; 4 Erasmus MC University Medical Center, Department of Neurology, Rotterdam, The Netherlands; 5 Erasmus MC University Medical Center, Department of Radiology & Nuclear Medicine, NL; 6 Erasmus MC University Medical Center, Department of Public Health, NL; 7 Rijndam Rehabilitation, The Netherlands; 8 Erasmus MC University Medical Center, Department of Rehabilitation, Rotterdam, NL

**Keywords:** value-based payment, stroke, payment reform, bundled payment, integrated care

## Abstract

**Background:**

To address issues related to suboptimal insight in outcomes, fragmentation, and increasing costs, stakeholders are experimenting with value-based payment (VBP) models, aiming to facilitate high-value integrated care. However, insight in how, why and under what circumstances such models can be successful is limited. Drawing upon realist evaluation principles, this study identifies context factors and associated mechanisms influencing the introduction of VBP in stroke care.

**Methods:**

Existing knowledge on context-mechanism relations impacting the introduction of VBP programs (in real-world settings) was summarized from literature. These relations were then tested, refined, and expanded based on a case study comprising interviews with representatives from organizations involved in the introduction of a VBP model for integrated stroke care in Rotterdam, the Netherlands.

**Results:**

Facilitating factors were pre-existing trust-based relations, shared dissatisfaction with the status quo, regulatory compatibility and simplicity of the payment contract, gradual introduction of down-side risk for providers, and involvement of a trusted third party for data management. Yet to be addressed barriers included friction between short- and long-term goals within and among organizations, unwillingness to forgo professional and organizational autonomy, discontinuity in resources, and limited access to real-time data for improving care delivery processes.

**Conclusions:**

Successful payment and delivery system reform require long-term commitment from all stakeholders stretching beyond the mere introduction of new models. Careful consideration of creating the ‘right’ contextual circumstances remains crucially important, which includes willingness among all involved providers to bear shared financial and clinical responsibility for the entire care chain, regardless of where care is provided.

## (1) Introduction

Healthcare systems around the world are currently facing the challenges of suboptimal (insight in) outcomes [1], fragmentation in care delivery [2], and increasing expenditures [3]. Being a leading cause of death and disability, stroke is one of the conditions facing all of these challenges [4,5]. Approximately 12.2 million strokes occur annually worldwide, of which 6.5 million result in death [4]. In addition, 143 million years of healthy life are lost each year due to stroke-related death and disability. Apart from its major impact on patients’ lives, stroke-related global costs (including long-term care and productivity loss) are estimated at 810 billion euro per year [4], a number that is expected to increase significantly due to population ageing [6]. Additionally, limited intersectoral collaboration further complicates the organization and delivery of integrated care [7].

A factor that is widely considered as contributing to these issues, are fee-for-service (FFS) payment systems that are used across many healthcare systems [8]. These systems reward providers for volume instead of value and obstruct providers in improving quality and coordination of care. As a response, stakeholders have increasingly experimented with value-based payment (VBP) models, including in stroke care [9]. In contrast to FFS, VBP models aim to facilitate and stimulate healthcare providers to realize the ambition of affordable, well-coordinated and high-quality integrated care from which patients should benefit [10]. Striving towards better Integrated care in this context includes the aim to improve outcomes for (chronic) health problems caused by stroke by overcoming fragmentation through linkage of provides over the care cycle [11], as well as enabling better alignment and collaboration between care sectors for better patient-relevant outcomes [12].

An increasingly applied form of VBP is bundled payment (BP). Instead of paying providers separately for each discrete care service provided (as in FFS), BP comprises a single, prespecified amount for providers assuming accountability for all services related to a certain medical condition, over a certain period. Ideally, BP covers all care that is necessary for treatment and management of the condition, regardless of where and by which provider care is provided when multiple providers are involved. To prevent a one-sided focus on efficiency and spending reduction, BP programs often also contain additional pay-for-performance incentives for high-quality outcomes. Although there is some evidence suggesting that BP has the potential to save costs while at least maintaining quality [13,14,15,16], convincing evidence on positive effects of BP on (stroke) care delivery is lacking [17,18,19].

It is well-established that, for a variety of reasons, the introduction of BP in practice is highly complex [20,21]. This complexity is illustrated by numerous examples of BP initiatives being terminated before even becoming operational, despite shared ambitions and significant efforts of involved stakeholders [20,22]. Additionally, BP programs are often confined, at least initially, to either hospital or primary care sectors [16], while shared cross-sector accountability for all necessary care for a condition is ultimately required to achieve integrated care [23]. Although some studies have focused on identifying (contextual) factors that may impact the introduction of BP programs [14], insight in these factors in different settings and particularly through which mechanisms the introduction of these programs is impacted remains limited [24].

Drawing upon realist evaluation principles, the goal of this study is to identify context-mechanism relations that facilitate or inhibit the introduction of an ongoing BP program in stroke care. This program, labelled PAying for Value in IntegrAted Stroke care (PAVIAS), was introduced on January 1st, 2019, in Rotterdam, The Netherlands. The program entails a BP contract with routine collection of patient-relevant outcomes and two-sided risk sharing between a large health insurance company and multiple healthcare providers (i.e., a large academic hospital and three rehabilitation care providers), aiming to facilitate and financially stimulate value improvement and integrated care delivery for ischemic stroke patients. Given the background and knowledge gaps described above, an in-depth analysis of the introduction of this program is expected to yield valuable insights and lessons because the program was successfully introduced and covers both short-term hospital care and longer-term rehabilitation care provided by multiple providers involved in the stroke care chain. Based on literature-informed interviews with directly involved stakeholders, we present an overview of context-mechanism relations with respect to the introduction of this BP program in stroke care and formulate key lessons for current and future (V)BP programs.

## (2) Methods

### (2.1) Study design

In this study we were particularly interested in providing information on how an outcome (i.e., the introduction of VBP) might generate different outcomes under different circumstances. Therefore, we drew upon the principles of realist evaluation (RE). In contrast to other forms of theory-driven evaluations [25], RE focuses on studying how and why interventions work or do not work by examining the specific mechanisms involved, such as changes in reasoning and behaviour, subject to different contextual influences. While RE thus aims to provide context-specific knowledge, implementation theory, for example, aims to identify generalizable principles for effective implementation [26]. Although the use of the RE-framework in health services research is relatively new, it is particularly suitable to evaluate complex interventions (such as the introduction of VBP programs) of which the success is dependent on both individual and social responses [27]. RE does not only aim to assess a particular outcome, but specifically also to identify relevant contextual factors and generative mechanisms (i.e., behavioural changes, reasoning, or perception of involved individuals) impacting this outcome. By identifying and synthesizing applicable context-mechanism-outcome (CMO) configurations, RE aims to create a profound understanding of the causal mechanisms (leading to an outcome) triggered by contextual influences [24].

Given our objectives, RE was selected as the suitable framework for this study. Drawing on RE principles, we investigated context-mechanism relations that influenced the outcome ‘introduction of the PAVIAS program’. This outcome was defined as the VBP contract having been signed and the program having commenced. Specifically, it refers to the stage where the program has been initiated on both an intra and inter-organizational level among providers. In this stage, stakeholders aim to establish data sharing systems, implementation of payment mechanisms and coordination among different healthcare professionals like care coordinators and clinicians.

To develop an initial understanding of the factors and mechanisms that influence the implementation of VBP programs, we first conducted a literature review on VBP implementation. The focus of this narrative review was to identify various CMO configurations regarding the introduction of VBP programs (in real-world settings). The review informed the design and contents of an interview guide and enabled us to contextualize our qualitative findings. Combining the terms value-based payment and implementation (and synonyms or strongly related keywords), we identified and synthesized key findings from thirteen included articles. From these articles, a total of fifteen CMO configurations were identified. Six articles focused on VBP in general (N = 6), five focused specifically on the introduction of BP (N = 5), and two concerned pay-for-performance (N = 2). A detailed description of the literature review and the identified CMO configurations is provided in Appendix 1.

The primary objective of this study was to examine the PAVIAS program through a case study approach. By conducting interviews with representatives from all stakeholders involved, we aimed to identify the contextual factors (C) that influenced the introduction of this program (O) and understand the mechanisms (M) by which these factors operated.

### (2.2) Data collection and analysis

We used documentation obtained from PAVIAS’ stakeholders to provide a detailed description of the program. This description is provided in Appendix 2. In total, thirteen non-public (internal) documents with information on the program’s goals, bundle definition, stakeholders involved, allocation of financial risk, and collection of data on outcomes and costs were obtained and reviewed [28]. Subsequently, we conducted ten semi-structured interviews with representatives of all relevant stakeholders. Respondents were purposively sampled based on their involvement in the introduction of PAVIAS and invited by email to participate. All invited individuals agreed to participate. Three respondents represented Erasmus University Medical Center (a neurologist, a project manager, and a professor of quality and outcomes of care); three respondents represented rehabilitation care provider Laurens (a director, a strategic advisor, and a care coordinator); two respondents represented health insurance company Zilveren Kruis Achmea (a senior care purchaser and a senior strategic advisor); one respondent represented rehabilitation provider Transmitt Rehabilitation (a director); and one respondent represented the Rotterdam Stroke Service (RSS, a director). The RSS is a regional cross-sector stroke care service with seventeen affiliated providers. Each of the above-mentioned provider organizations were already affiliated with the RSS prior to the introduction of PAVIAS.

We created an interview guide (Appendix 3) using the CMO configurations derived from literature (see also Appendix 1), aiming to expand, refine and revise these configurations during the interviews.

The interviews consisted of two parts. In the first part, respondents were asked open-ended questions to gather information about contextual factors and associated mechanisms. Follow-up questions were then asked based on their responses to explore the importance of specific context factors, as well as how, why, and for whom they were relevant. This allowed for the examination of mechanisms before proceeding to the second part of the interview. In the second part, existing propositions on CMO configurations were tested. This order was chosen to minimize bias from confirming predetermined mechanisms and to mitigate risks caused by the interview process.

Before the interview, the first part of the interview guide was emailed to respondents to allow them to prepare and reduce recall bias. Informed consent for recording the interview and using the data for the study was obtained from all respondents prior to the interviews. Each interview began with the interviewer (the lead author) and respondent reaching a consensus on the interview goals, defining key terms, and clarifying the respondent’s perceived role in the PAVIAS program and its introduction. The respondents were then asked open-ended questions about the introduction process, factors that facilitated or hindered the introduction, and the reasons behind them. Questions focused on the perceived positive or negative aspects of the program’ introduction and were asked about the most important factors, main barriers, how these barriers were partially overcome, and lessons learned. All interviews were conducted in an end-to-end-encrypted video-meeting using Microsoft Teams software. The average interview duration was 50 minutes (range 40–70 minutes).

The audio-recordings were transcribed verbatim and thematic analysis was performed on the transcripts. The lead author coded the interview data, which resulted in an initial list of 42 codes each referring to a context-mechanism relation that was believed to have impacted the introduction of the PAVIAS program. This list was then discussed and adjusted in several meetings with four authors, eventually leading to 28 codes each representing a specific CMO configuration. Finally, these codes were pragmatically grouped in six overarching themes based on similarity in mechanisms triggered by context factors. For example, the theme ‘Trust, relations, and support’ contains context factors that triggered mechanisms related to feelings of shared commitment, fear, and/or scepticism.

Following the coding process, all respondents were approached for a member check [29]. Specifically, respondents were sent the coded data based on their interview and were asked whether these codes accurately reflected their viewpoint and perception. Five respondents responded, of which two suggested minor additions. All interview data were analysed using Atlas.ti version 9 software.

## (3) Results

### (3.1) Factors influencing PAVIAS’ introduction

This section discusses all context-mechanism relations that were identified as having been influential during the introduction of PAVIAS (see Table 1 for an overview). Below, these relations are discussed under six overarching themes, with identified contextual factors and the corresponding mechanism(s) in italics and labelled as C_n_ and M_n_.

**Table 1:** Identified context-mechanism relations that were mentioned by respondents as having impacted the introduction of the PAVIAS program in Rotterdam, the Netherlands.


THEME	CONTEXT FACTOR AND DESCRIPTION		MECHANISM(S) DESCRIPTION (MECHANISM #)

**Goals & motivation**	C1	Across stakeholders, the main goals and origin of motivations for initiating the program were generally overlapping.	*Feelings of frustration with the current situation (M1)*, *feeling a shared sense of urgency for change (M2)*, *the potential benefit (i.e., better value for patients or more knowledge on VBP) worth the time and effort (M3)*

	C2	Although respondents generally had similar overarching goals, they had substantially different views on how to achieve and operationalize these shared goals.	*Perceived tension between short and long-term goals (M4)*, *demotivating conflicts of interest that undermine a shared rationale (M5)*

	C3	Some respondents expressed *uncertainty about whether the introduction of the program would substantially improve value in the short run* due to limited patient volumes and time required to make significant changes to healthcare delivery.	*Perceived tension between short and long-term goals (M4)*, *scepticism about meaningful change in a reasonable time (M6)*

	C4	Lacking evidence on positive effects of VBP and limited experience with integrated payment hardly affected their motivations to contribute to the program.	*Feelings of frustration with the current situation (M1)*, *feeling a shared sense of urgency for change (M2)*

	C5	Motivational leadership of individuals from different organizations was identified as a major contributing factor. Such leadership entailed setting deadlines, showing clear dedication to meet these deadlines, and persuasion of other people.	*feeling of having a shared commitment to make the program work (M7)*

**Trust, relations & support**	C6	*The existence of good historic working relations and pre-existing trust among stakeholders with a good reputation was a crucial contributor to the introduction of the program*.	*Feeling comfortable in making investments (M8), having a feeling of ‘being in it together’ (M9)*

	C7	Strong organizational support was an often-mentioned facilitator, although some respondents representing the hospital added that more pro-active support could have prevented delay.	*Feeling comfortable in making investments (M8), Having limited fear of (severe) repercussions during trial and error (M10)*.

	C8	All respondents mentioned some degree of conflicting interests between and within organizations.	*scepticism about each other’s motives (M11)*, *perceived suboptimal inter- and intra-organizational relationships (M12)*

	C9	There appeared to be a lack of a shared responsibility for (the costs of) all care in the bundle. Some stakeholders only considered responsibility for care delivered by their own organizations, whereas others (n = 3) stressed the importance of joint responsibility for all care in the bundle, including care provided by other organizations.	*high perceived importance attached to autonomy (M13)*, *perceived loss of control over responsibilities (M14), professional obstination (M15)*

**Design of VBP contract**	C10	The introduction of the program was experienced to be complex. in dealing with this complexity, the decision to make an outline agreement (instead of attempting to reach consensus on a detailed and complex contract that accounts for all possible contingencies) was beneficial.	*Perceived complexity and experienced control over the program (M16)*

	C11	The choice for a multi-year contract with no accountability for financial losses in the first year was identified as a contributing factor.	*Reduced reluctance and uncomfortable feelings of being exposed to too much risk from the outset (M17)*

	C12	Although the reluctance to take on financial risk was generally low (in part because stroke-related revenue was relatively small for most stakeholders), respondents did mention that the degree of financial risk under the program varied heavily among stakeholders.	*The potential benefit (i.e., better value for patients or more knowledge on VBP) (not) worth the time and effort (M3)*, *Loss or gain of interest in the program (M18)*.

**Regulatory compatibility**	C13	The compatibility of the BP contract with the existing FFS reimbursement rules and billing system facilitated the introduction of the program	*Limit the perceived complexity and enhance experienced control over the program (M16)*

	C14	Existing privacy and anti-trust legislation was a barrier, especially with respect to data exchange among competing organizations. This barrier was partly overcome by involving a trusted third party (TTP) for data definition, accumulation, and comparison	*Perceived complexity and enhance experienced control over the program (M16), reduced experienced possibilities for care coordination among stakeholders (M19)*, *scepticism about the possibilities for improving care (M20)*

**Resource management**	C15	The degree to which resources were made available and the level of leadership was generally proportional to the size of the respective stakeholder organizations	*feelings of fairness (M21)*, *perceived equality in workload (M22)*

	C16	A lack of continuity in personnel and project groups was a barrier leading to delays (e.g., people in key positions leaving to other employers, insufficient feedback among different project subgroups, premature disbandment of these groups without follow-up)	*Perceived support and cooperation (M23)*, *feelings of demotivation (M24)*

	C17	Insufficient human and financial resources frustrating effective program management was identified as a barrier	*High perceived workload (M25), feelings of stress (M26), and feelings of dissatisfaction (M27)*

**Data management & monitoring**	C18	The involvement of a trusted third party (TTP) for data management was mentioned as an important contributing factor for making shared data definitions, financial metrics, accumulation of data, and providing insights into achieved outcomes and costs	*Perceived complexity and experienced control over the program (M16)*, *confidence and trust in the validity of data (M28)*


### (3.2) Goals and motivation

Across stakeholders, *the main goal and origin of motivation for initiating the program were generally overlapping* (C_1_), albeit on a coarse level. Shared goals were described as: striving towards higher value of stroke care through defragmentation, improvement of interprofessional communication, and contributing to the value-based healthcare (VBHC) evidence base. Motivations among stakeholders were driven by *profound feelings of frustration with the current situation* (M_1_) in which progress was perceived to be hampered by the predominant FFS payment system. Relatedly, they were *experiencing a shared sense of urgency for change* (M_2_). Furthermore, most respondents (n = 7) noted that *the potential reward was perceived as being worth the time and effort* (M_3_). As one respondent outlined:

“*It was not only a personal drive to improve care. In general, the scientific substantiation for value-based healthcare is thin. In that regard I am willing to contribute to innovative programs like this. It is important that we contribute to science by doing so.” – Respondent 5*

Although stakeholders generally had similar overarching goals, according to the respondents they had *substantially different views on how to achieve and operationalize these shared goals* (C_2_), which appears to have contributed to *perceived tension between short and long-term goals* (M_4_) and *demotivating conflicts of interest that undermine a shared rationale* (M_5_). For example, concrete plans or agreements on how to improve value were absent or ill-defined in advance, although sometimes – as mentioned by two respondents – this was a deliberate strategy to prevent delay by too much focus on specific goals about which reaching consensus may be difficult (see also C_10_). When asked whether goals were concretized prior to introduction, one respondent replied:

*“Yes and no. Defining goals is an iterative process. I think it is naïve to assume that the specific stakeholder goals align. But I think it is realistic to assume that aggregated goals are aligned, and that should be emphasized. Whenever things get more concrete, misalignment becomes more likely. It’s a process of interaction.” – Respondent 6*

Nevertheless, the fact that specific goals or operationalizations thereof were not always shared may have led to conflicts among or within stakeholder organizations (see also the theme Trust, relations, and support). An example is the explicit goal of the insurer to limit spending, while some providers (n = 2) wanted to spend more to improve care. Remarkably, all respondents except the representatives from the insurer expressed that they did not see a (short-term) financial benefit from participating in the program because stroke patients are seen as an unprofitable population, or they expected to incur more (short-term) costs due to allocation of resources needed for introducing the program.

Some respondents (n = 3) expressed *uncertainty about whether the introduction of the program would substantially improve value in the short run* (C_3_) due to limited patient volumes and time required to make significant changes to healthcare delivery (see also the theme Resource management). Triggered mechanisms that negatively impacted motivations in this respect were *perceived tension between short and long-term goals* (M_4_) and *scepticism about whether meaningful change could be realized in a reasonable time* (M_6_).

For most respondents (n = 9), *lacking evidence on positive effects of VBP and limited experience with integrated payment hardly affected their motivations to contribute to the program* (C_4_); as noted by the respondents, BP-contracts such as PAVIAS are new to the Dutch healthcare system and evidence from other countries is likely to have limited applicability in the Dutch context. Mentioned mechanisms were again *profound feelings of frustration with the current situation* (M_1_) and *a shared sense of urgency for change* (M_2_). As one respondent summarized:

*“I had zero experience with VBP, and others had very little. However, this program was one of a kind anyway.” – Respondent 9*

When asked about the factor(s) that contributed most to the introduction of the PAVIAS program, most respondents (n = 7) mentioned *motivational leadership of individuals from different organizations* (C_5_). Such leadership entailed setting deadlines, showing clear dedication to meet these deadlines, and persuasion of other people to introduce the program, all of which bolstered *the feeling of having a shared commitment to make the program work* (M_7_).

### (3.3) Trust, relations, and support

All respondents acknowledged that *the existence of good historic working relation and pre-existing trust among stakeholders with a good reputation* (C_6_) was a crucial contributor to the introduction of the program. As a result, stakeholders *felt comfortable in making investments* (M_8_) and experienced *a feeling of ‘being in it together’* (M_9_). As one respondent explained:

*“An important element was pre-existing trust. The Rotterdam Stroke Service, for example, already exists for 25 years. We have been working together intensively for a long time and it was not the first time we were gathered around the table when we conceptualized this program. We all expressed a desire to do this together.” – Respondent 5*

*Strong organizational support* (C_7_) was an often-mentioned facilitator, although some respondents representing the hospital added that more pro-active support could have prevented delay. As a result of the perceived support, stakeholders felt *comfortable in making investments* (M_8_) and had *limited fear of (severe) repercussions during trial and error* (M_10_). As one respondent exemplified:

*“Management made it possible by not blocking anything, although I think that things would have gone quicker if the board would have had an attitude like ‘we back your plans, and we will make efforts to expedite the process.”’ – Respondent 9*

All respondents did mention some degree of *conflicting interests between and within organizations* (C_8_). They noted that this contributed to *scepticism about each other’s motives* (M_13_) and *perceived suboptimal inter- and intra-organizational relationships* (M_14_), which shifted focus away from shared goals. As explained by one respondent:

*“In our organization, one board member is responsible for VBHC whereas another is responsible for IT or finance, while you need all those disciplines at the table. Unfortunately, that proved to be very hard due to differing degrees of priority given to the program by the different board members.” – Respondent 9*

In addition, there appeared to be a *lack of a shared responsibility for (the costs of) all care in the bundle* (C_9_). Some stakeholders (n = 4) only considered responsibility for care delivered by their own organizations, whereas others (n = 3) stressed the importance of joint responsibility for all care in the bundle, including care provided by other organizations. Mentioned mechanisms were *a high perceived importance attached to autonomy* (M_13_), a *perceived loss of control over responsibilities* (M_14_), and *professional obstination* (M_15_). One respondent noted:

*“An important factor for this program to be successful is that you must let go of some autonomy to bear shared responsibility. You must be willing to compromise.” – Respondent 5*

### (3.4) Contract design

The introduction of the program was experienced to be complex. Some respondents (n = 3) noted that in dealing with this complexity, *the decision to make an outline agreement* (instead of attempting to reach consensus on a detailed and complex contract that accounts for all possible contingencies) *was beneficial* (C_10_). This was mentioned to *limit the perceived complexity and enhance experienced control over the program* (M_16_). As one respondent explained:

*“An important lesson I’ve learned is that too much discussion about financial and contractual details may be a cause of failure for such programs.” – Respondent 10*

Additionally, *the choice for a multi-year contract with no accountability for financial losses in the first year* (C_11_) was mentioned (n = 6) as a contributing factor. This reduced *reluctance and uncomfortable feelings of being exposed to too much risk from the outset* (M_17_) among stakeholders. As one respondent described:

*“For the first year we agreed there would be no shared losses if outcome measures were collected and reported. Accountability for losses would go into effect in a later stage. I think that such ‘phasing’ contributed to mitigating reluctance among providers.” – Respondent 4*

Although the reluctance to take on financial risk was generally low (in part because stroke-related revenue was relatively small for most stakeholders), respondents (n = 3) did mention that *the degree of financial risk under the program varied heavily among stakeholders* (C_12_). In turn, *the experienced potential benefit of participating in the program may not have been perceived as being worth the effort to a similar degree* by all stakeholders (M_3_). Depending on the extent to which this is the case, stakeholders *might lose or gain interest* in the program (M_18_).

### (3.5) Regulatory compatibility

Respondents from the involved insurer (n = 2) noted that *compatibility of the BP model with the existing FFS reimbursement rules and billing system* facilitated the introduction of the program (C_13_). Compatibility in this context means that the existing FFS architecture was left intact and that FFS claims made during the year would be retrospectively reconciliated with the virtual bundle price to determine savings or losses. The fact that the contract could be executed under existing payment regulations *limited the perceived complexity and enhanced control over the program* (M_16_) One respondent summarized this as follows:

*“Our principle was that this program should be compatible with the current reimbursement system. I truly believe that letting that principle go would be a recipe for disaster. Because of this, complex interventions, such as standardization of financial systems among stakeholders were not necessary.” – Respondent 1*

In contrast, all respondents viewed existing *privacy and anti-trust legislation as a barrier, especially with respect to data exchange among competing organizations* (C_14_). Mentioned mechanisms were the *perceived complexity and loss of control over the program* (M_16_), the *reduced experienced possibilities for care coordination among stakeholders* (M_19_), and *scepticism about the possibilities for improving care* (M_20_) for which free exchange of data is deemed crucial. This barrier was partly overcome by involving a trusted third party (TTP) (see also the theme Data management & monitoring). One respondent summarized:

*“It is very bothersome that we must adhere to rules that don’t benefit patients. I get why legislation and regulations exist, but these are insufficiently geared towards healthcare trends and coordinating care around patients” – Respondent 5*

### (3.6) Resource management

According to all respondents, *the degree to which resources were made available and the level of leadership was generally proportional to the size of the respective stakeholder organizations* (C_15_). Respondents mentioned that this contributed to *feelings of fairness* (M_21_) and *perceived equality in workload* (M_22_).

However, multiple respondents (n = 6) identified *a lack of continuity in personnel and project groups* (C_16_) as a barrier leading to delays. Examples are people in key positions leaving to other employers and premature disbandment of project groups without follow-up. Mechanisms triggered by this factor as mentioned by the respondents were *insufficient perceived support and cooperation* (M_23_) and *feelings of demotivation* (M_24_). As one respondent noted:

*“A clear barrier was that employees come and go during the introduction of such a program. Every time that happens you must bring new people up to speed. That significantly delayed the process.” – Respondent 6*

*Insufficient human and financial resources frustrating effective program management* (C_17_) was also mentioned (n = 3) as a barrier. Mentioned mechanisms were a *high perceived workload* (M_25_), feelings of *stress* (M_26_), and *feelings of dissatisfaction* (M_27_). One respondent remarked:

*“Every healthcare professional is already trying hard and cannot spare time to work on this program. That could be achieved by reorganizing and making one person responsible for a certain task, but that sort of creative thinking is not happening yet. – Respondent 9*

### (3.7) Data management & monitoring

Six respondents mentioned the *involvement of a TTP for data management* (C_18_) as a contributing factor. Three reasons were provided for this. First, it reduces the perceived likelihood of data manipulation. Second, a TTP partly overcomes regulatory issues such as exchange of sensitive personal data (see also the theme Regulatory compatibility). Third, the TTP assisted in overcoming the challenge of defining shared and standardized quality and financial metrics, which were deemed crucial by all stakeholders. Triggered mechanisms were *reduced perceived complexity and increased control over the program* (M_16_) due to centralized data management as well as *confidence and trust in the validity of data* (M_29_).

## (4) Discussion

### (4.1) Summary and discussion of main findings

In this study we identified and analysed context-mechanism relations that influenced the introduction of a VBP program in integrated stroke care in Rotterdam, the Netherlands. Using literature-informed semi-structured interviews with representatives from key stakeholders, 18 context factors and 28 related mechanisms were identified. Most context-mechanism relations found in literature were also identified by at least one interview respondent to some degree. Below, we discuss the key findings and derive lessons for the introduction of future VBP programs.

Several factors clearly contributed to the program’s introduction. First, the good pre-existing working relations and trust among stakeholders were identified as important contributors. The intensive collaboration required for cross-sectoral VBP programs such as PAVIAS requires a solid foundation for stakeholders to feel comfortable with investing in payment and delivery system reform. This factor was also identified in previous studies as a crucial determinant of the success or failure of VBP implementation [20,30,31]. A second, related facilitator was the existence of strong motivation for change among all stakeholders due to shared dissatisfaction with the status quo in which patients could often not receive appropriate care in the shortest time frame. This motivation was also further bolstered by motivational leadership of key-individuals from different organizations.

Third, respondents highlighted the decision to build the new payment model on the existing FFS architecture as a key factor that likely has prevented many demotivating issues and delays that would be involved with replacing the current payment and billing system. This factor was also often mentioned in the literature, sometimes even as having contributed to the failure of VBP programs [14,21,22,30,32,33,34,35]. Fourth, although the introduction of the program was considered complex, a contributing factor was the use of an outline agreement reducing the chance of difficult, demotivating discussions on contractual details. However, this strategy involves a trade-off between short-term progress and potential conflicts in the longer run about specification and operationalization of overarching goals. Finally, the involvement of a TTP was mentioned as a contributor that facilitated data monitoring and management across different providers.

Several key inhibiting CM-relations are also worth discussing. Although these apparently did not prevent the eventual introduction of the program, they did cause issues and delays, and might hamper future success if not addressed. First, although all stakeholders are willing to take on financial risks, reaching agreement on financial-risk sharing remains an unresolved issue mainly due to differences in the proportion of stroke-related revenue relative to total revenue. This has negatively impacted a balanced interest in the program, with stakeholders with a larger proportion potentially opting-out due to too high perceived financial risk relative to other participating providers. This issue was also often-mentioned in literature, though without insight in related mechanisms [14,20,21,22,30,31,33]. Second, discontinuity in human and financial resources was identified as an important barrier. This has led to demotivating delays, especially because it coincided with organizational management sometimes being labelled as ‘passive’ in terms of limited investment in propagating commitment to the program across all organizational layers. This latter barrier has been mentioned before [36]. A third important barrier was insufficient willingness among stakeholders to let go of professional or organizational autonomy. Relatedly, stakeholders were not (fully) willing to bear shared responsibility for patient outcomes and spending in the entire stroke care chain. These two issues stand in stark contrast to the overarching goal of realizing integrated care and therefore form a major challenge to be addressed moving forward. Fourth, friction among stakeholders caused by tension between short- and long-term goals were identified as a barrier. Although goals among stakeholders on an aggregated level were similar (e.g., increasing value for patients), there were – for example – conflicting ideas on whether this goal should be reached by spending more or by spending less. A final identified barrier was limited access to real-time data for effective feedback and input for improving care delivery processes.

In contrast to the findings of prior work [21,34,35,37,38,39], the limited evidence on positive effects of VBP programs has had remarkably limited influence on the program’s introduction. The reason as described by the respondents is that such evidence, which mainly comes from other countries, has limited applicability to the Dutch context with its unique features. Another possible reason is that PAVIAS can be characterized as a pilot program in which stakeholders are ‘learning by doing’ in a safe environment for experimentation. This contrasts with the more definitive nature of VBP programs evaluated in other studies, in which lacking evidence on positive impact was often identified as barrier.

### (4.2) Lessons for future VBP programs

Our study yields several key lessons for VBP reform involving collaboration between multiple provider organizations, particularly in the field of stroke care but likely also for other conditions.

First, good trust-based working relations between all intended contract-partners are ideally established *prior* to introducing a new payment model. This is expected to significantly increase stakeholder acceptance and comfort in making joint investments, as well as assist in respecting each other’s (often differing) motives and interests.

Second, defining clear goals for the short and long run (e.g., what exactly needs to change, who is involved, how can goals be achieved, what are the intended outcomes) among and within stakeholder organizations is important. This may prevent future conflicts of interest and demotivating discussions which could ultimately result in program failure.

Third, involving a TTP for data management is advisable. Although a TTP is unlikely to be able to match the benefits of a fully integrated electronic health record (which governmental bodies often disallow), it can assist in the collection of data that are trusted by all stakeholders, overcoming potential legal issues regarding data exchange, and standardization of quality and financial metrics.

Fourth, to enable representative bundle contents, it is recommended to accumulate several years of patient and financial baseline data *prior* to introduction. Such data would increase the likelihood of the bundle price accurately reflecting the costs of the current standard of care as it can be based on the most recent data. Additionally, it would better enable rigorous evaluation of the impact on outcomes and spending.

A fifth lesson is that long-term commitment to the program of all involved organizations is crucial. Stakeholders should explicitly assign a high priority to the program, which includes showing willingness to allocate sufficient resources to it, forgo some organizational and professional autonomy, and accept shared responsibility for spending and quality outcomes beyond their full control.

Sixth, in designing the payment contract it is advisable to allow time for providers to adapt to integrated payment and bearing of financial risk, for example by a ‘soft’ replacement of FFS using a retrospective payment methodology and without downside risk in the first year(s).

Finally, payment and delivery system reform are clearly not finished after signing a contract. It is crucial to acknowledge that additional steps and considerations are necessary for successful reform. For instance, in the current contract, the decision was made to exclude primary care as a domain due to the complexity already involved with the existing number of stakeholders. While too many variables could potentially lead to failure of VBP programs, the inclusion of primary care is desirable for future expansion. Therefore, successful reform requires long-term commitment from all stakeholders, in which healthcare professionals ultimately have a key position. This requires time, resources, a constructive regulatory environment, and inspiring leaders as well as continuous efforts in making progress explicit, which is crucial for keeping professionals engaged in realizing the goal of increasing value for patients.

### (4.3) Strengths and limitations

A key strength of this study is that it is one of the very few that examined both contextual factors *and* related mechanisms regarding the introduction of a VBP program for multiple care provider organizations. Insight into how context impacts complex interventions and through which generative mechanisms is valuable to better understand the causal path to certain outcomes. Another strength is that we drew upon realist evaluation principles and used literature on the introduction of VBP programs for in-depth interviews with representatives from *all* relevant stakeholders, which allowed us to provide a comprehensive picture of influencing factors and mechanisms.

However, several limitations should also be mentioned. First, we only focused on CM-relations regarding the *introduction* of the PAVIAS program. Further research focusing on uncovering CM-relations that impact its further implementation and success in terms of changes in patient outcomes and spending is required to assist providers and policymakers further in realizing successful payment reform. Second, although we believe our results provide valuable insights for (future) VBP programs for stroke as well as other conditions, the generalizability of our findings to other (inter)national settings is uncertain. Third, the reliance on retrospective responses from the sampled group of respondents may have biased our results due to their (shared) perception of success and the influence of reflecting with hindsight on their experiences. Future research could explore the perspectives of individuals who were not initially involved in the development and introduction of the VBP contract – such as patients, managers, clinicians, and other caregivers – but who are affected by it in practice. Finally, we acknowledge that the inherently subjective nature of defining and delineating contextual factors and generative mechanisms may to some degree have impacted the validity and reliability of the identified C-M configurations. In this paper we used the definition that context refers to observable surrounding conditions and factors that influence the implementation of an intervention, while mechanisms represent the unobservable underlying processes and reasonings through which the context factors result in the outcome.

## (5) Conclusions

Several important preconditions and facilitators were in place that aided the introduction of a value-based payment program in integrated stroke care. Among the most important factors were good pre-existing and trust-based working relations, a strong motivation for change among all stakeholders due to shared dissatisfaction with the status quo, motivational leadership to keep everyone engaged and committed, simplicity and regulatory compatibility of the payment contract, and the involvement of a trusted third party for data monitoring and management. Despite substantial barriers both within and between stakeholder organisations, these did not prevent the program’s introduction.

Nonetheless, going forward several issues will need intensive attention if the program is to fulfil its promise of facilitating integrated high-value stroke care. These issues include friction among stakeholders caused by tension between short- and long-term goals, unwillingness to let go of some professional or organizational autonomy, discontinuity in available financial and human resources, and limited access to real-time data for effective feedback and input for improving care delivery processes. Finally, and most crucially, all providers should be willing to bear shared financial and clinical responsibility over the entire stroke care cycle, regardless of where care is provided.

## Data accessibility statements

This study brought together data obtained upon request and subject to restrictions from several different sources. The database is not publicly available due to the (politically and financially) sensitive nature of the data.

## Additional Files

The additional files for this article can be found as follows:

10.5334/ijic.7566.s1 Appendix 1. CMO configurations derived from literature.

10.5334/ijic.7566.s2 Appendix 2. Integrated care case description.

10.5334/ijic.7566.s3 Appendix 3. Interview guide.
