# Supplementary material for: Factors Influencing the Introduction of Value-Based Payment in Integrated Stroke Care: Evidence from a Qualitative Case Study
Source: Int J Integr Care. 2023 Aug 10;23(3):7. doi: 10.5334/ijic.7566 (PMC10437137; doi:10.5334/ijic.7566)
Supplement: Appendix 1. — CMO configurations derived from literature. [file ijic-23-3-7566-s1.pdf]

Appendix 1. CMO configurations identified from literature

Data collection and analysis

The aim of the literature review was to create an overview of CMO configurations that reflect the complex dynamics of the introduction of VBP. Both conceptual and empirical peer-reviewed articles written in English about context factors and mechanisms related to the introduction of VBP programs were searched in MEDLINE. We applied the following search string including MeSH terms: ((Alternative payment model/method*) OR Bundled payment OR Value based payment AND (Barrier* OR Facilitat* OR Challeng* OR Factor* OR Design* OR Implement* OR Evaluat*)). We were particularly interested in articles reporting on VBP programs in stroke care, but we also included articles on VBP in general and for other conditions because we expected these to potentially contain valuable insights relevant to our outcome. After the initial screening of titles and abstracts (N=345), 59 full-text articles were obtained and evaluated for inclusion by two authors. Studies that solely evaluated VBP programs beyond the introduction phase (e.g., on their impact on spending and/or quality) were excluded. In total, thirteen studies were included, of which seven were literature reviews and six were either conceptual or case studies. These articles were then assessed for possible CMO relations, with the O being defined as 'introduction of VBP'. While identified contextual factors were always mentioned literally in these articles, mechanisms were typically not explicitly described. In these cases, mechanisms (i.e., behaviours, reasonings and perceptions triggered by contextual factors) were inferred by the lead author.

Below and in Figure 1, we present an overview of the CMO configurations that resulted from the narrative review. The configurations are described in order from most to least mentioned in the thirteen identified studies. In general, the analysed literature reports more extensively on barriers to VBP introduction than on contributing factors. Often-encountered barriers were legislative and regulatory issues, disagreement among stakeholders on financial-risk sharing, lacking evidence on the benefits of integrated payment, and lacking leadership and trust among stakeholders. Additionally, lacking electronic information systems and difficulty in establishing standardized definitions of financial and quality metrics across organizations were often mentioned as challenges. Associated mechanisms were often related to perceived complexity of the program and its introduction, reluctance to take on (financial) risks, and uncertainty about whether rewards are worth the extra time and effort.

(A) An often-encountered barrier was (state) legislation and regulations complicating the introduction of VBP programs[1]'[2]'[3]'[4]'[5]'[6]'[7]'[8]. Examples are privacy and antitrust laws that

complicate collaboration and exchange of financial and medical information among involved providers, and incompatibility of the current (FFS) billing system with integrated payment. These issues impact the initial possibility of introduction of VBP programs, as well as influence of perceived stakeholder control over and interest in such programs. In turn, these mechanisms heavily influence the chances that VBP programs can be successfully introduced.

(B) Another often mentioned factor was disagreement between stakeholders on financial-risk sharing [1]'[9]'[2]'[3]'[5]'[8]'[10]. A mechanism that likely underlies these difficulties are feelings of ‘uncertainty’ and ‘unfamiliarity’ with new payment systems, as well as ‘reluctance’ to take on (more) financial risk. In turn, this negatively impacts stakeholder motivation and subsequently participation rates in VBP programs.

(C) Lacking evidence on the benefits (financial or medical) of integrated payment was mentioned as a contextual factor in five articles[2]'[6]'[7]'[11]'[12]. In turn, this might negatively influence stakeholders ‘(inferred) motivation and acceptance’ of VBP programs.

(D) There was wide variation in demonstrated level of effort between stakeholders, which was found to often frustrate progress[1]'[9]'[6]'[8]'[10]. Literature however mentions that extraordinary efforts made by key individuals were a facilitator which drove progress. A likely underlying mechanism is ‘varying perception among stakeholders of the extent to which rewards are worth the (extra) time and effort’.

(E) Varying degree of trust between stakeholders was mentioned several times as an important contextual factor influencing VBP introduction[9]'[8]'[10]. Related mechanisms might include a shared commitment among stakeholders to learn and/or having a feeling of being ‘in it together’. On the other hand, a lack of trust might be fostered due to scepticism about each other’s motives, negative inter- and intra-organizational dynamics.

(F) Lacking electronic information systems were mentioned in three articles as being a factor that frustrates effective program management in all phases of VBP introduction and implementation[9]'[6]'[8]. Lacking electronic information systems make it challenging for stakeholders to sufficiently exchange financial and/or medical data which impacts the visibility of (possible) impact which in turn demotivates and fosters uncertainty.

Classification: Internal

(G) Difficulty in establishing standardized definitions of financial and quality metrics across stakeholders was mentioned as a contextual factor that complicated efficient cross-organizational collaboration[1],[6]. A likely related mechanism is a lack of perceived need or willingness to invest in data-uniformity across stakeholders because they do not see the goal or do not want to lose autonomy, which in turn complicates the introduction of VBP programs.

(H) Two articles mentioned the issue that the more intensive collaboration involved with introducing VBP programs can be (too) time consuming[3],[5]. A related mechanism might be that stakeholders (un)consciously make a cost-benefit analysis and have doubts whether the potential reward would be worth the relatively large amount of additional invested time. As a result, this could make contributing to such programmes less interesting for stakeholders.

(I) One article raised the point that given that multimorbidity is expected to increase, concerns exist on if and how VBP programs should use different or additional (integrated) payments for patients with multi-morbidity[7]. This contextual factor likely influences the perceived 'ease of and control over' the program by stakeholders because a lack of financial reimbursement for more expensive patients might increase 'reluctance for financial risk and feelings of uncertainty'. In turn, this is expected to impact stakeholder' interest in contributing to program.

(J) Unclear division of medical or financial responsibility and accountability caused by VBP programs was mentioned in one article as a contextual factor impacting introduction[7]. Since care from different providers is aimed to be delivered in an integrated manner in VBP programs that include multiple types of providers, this might cause confusion on which professional or entity holds (legal) financial or medical end-responsibility. This may coincide with a perceived loss of organizational or professional autonomy which might shift focus away from shared end-goals.

(K) Experienced difficulty with possible changes in historical working relations with other stakeholders (resulting from VBP introduction) was mentioned as a barrier in one article[6]. Again, this is likely caused by stakeholders (un)consciously making a cost-benefit analysis and being hesitant regarding whether the potential reward would be worth the extra effort these changes might pose.

(L) One article mentioned possible data manipulation (i.e., gaming) as a contextual factor that might be exacerbated by the introduction of VBP programs[1]. This would, for example, mean that

Classification: Internal

providers will focus on achieving excellent scores on data relevant to (financial) rewards in their VBP program (i.e., not necessarily to what matters to patients), while neglecting other patient-relevant parameters. Related mechanisms could include low confidence in improved value of care due to the program and/or low intrinsic motivation due to such behaviour.

(M) Another limiting contextual factor mentioned in one article concerned the potential limitation of patient choice between providers[1]. If, for example, the care provider of choice is not included in the VBP program patients might not receive reimbursement from their insurer if they choose to visit these providers, resulting in a limit on 'freedom of choice' between providers for patients. Likely related mechanisms include fear of losing freedom of choice among patients and resistance among providers who might lose revenue as a result. The latter likely impacts interest in the program.

(N) A perceived lack of knowledge of / experience with how to effectively introduce a VBP program in practice was mentioned as a barrier once[13]. This lack is likely to result in delays and issues caused by inefficient program management and possible misalignment of interests among stakeholders.

Inferred mechanisms underlying this context-factor might be feelings of demotivation and dissatisfaction.

(O) Perceived lack of support from organizational management was mentioned to negatively impact the success rate of health care improvement projects in general, and vice versa[14]. Mechanisms that are likely subjected to this context were perceived support and cooperation and feelings of demotivation.

A. (In)compatibility of financing and privacy legislation with integrated financing[1]′ [2]′ [3]′ [4]′ [5]′ [6]′ [7]′[8].
B. Disagreement on financial risk sharing between stakeholders[1]′ [9]′[2]′[3]′[5]′[8]′[10].
C. Lack of evidence on benefits of integrated financing[2]′ [6]′ [7]′ [11]′[12].
D. Varying level of effort and leadership between stakeholders[1]′[9]′ [6]′ [8]′[10].
E. (Lack of) trust among stakeholders[9]′ [8]′[10].
F. Lacking electronic information systems[9]′ [6]′[8].
G. Difficulty in defining standardized (quality) definitions[1]′[6].
H. Necessary collaboration for VBP contract more time consuming[3]′[5].
I. Multitude of separate financing programs for patients with multi-and comorbidity[7].
J. (In)adequate division of medical and financial responsibility and accountability[7].
K. Difficulty with alteration of historic working relations due to VBP[6].
L. Possibility of data-manipulation (gaming)[1].
M. Potential limits on patient ‘freedom of choice’[1].
N. Lack of knowledge/experience of stakeholders with VBP[13].
O. (Lack of) support from organizational management[14].

A. Makes incentive (im)possible to implement, influencing complexity, perceived control, and interest.
B. Replacement of FFS leads to unfamiliarity, reluctance, and feelings of uncertainty concerning financial risk bearing.
C. (Lower) stakeholder acceptance of incentive due to intrinsic motivation (need for reform) shared rationale among providers.
D. Potential reward not perceived as worth time/effort
E. (Lack of) joint stakeholder intent due to shared sense of urgency and desire for reform.
F. (In)effective program management due to (in)visible impact that (de)motivates and fosters (un)certainty.
G. Insufficient consensus on rational investment/need for administrative and clinical data uniformity
H. Reward not perceived as worth the extra effort (cost-benefit).
I. Reluctance for financial risk and feelings of uncertainty’
J. Shifts focus away from end goal at both an organizational and individual level (perceived sense of autonomy)
K. Reward not perceived as worth the extra effort (cost-benefit).
L. Influences perceived credibility, predictability, and confidence.
M. Feelings of fear among patients, reluctance among providers.
N. Feelings of demotivation and dissatisfaction.
O. Perceived support and cooperation and feelings of demotivation.

Context

C

Introduction of Value-based payment

Figure 1 - CMO configurations synthesized from narrative literature r

1. Struijs J, de Vries EF, Baan CA, van Gils PF RM. Bundled-Payment Models Around World: How They Work, Their Impact | Commonwealth Fund [Internet]. 2020 [cited 2020 Nov 28]. Available from: https://www.commonwealthfund.org/publications/2020/apr/bundled-payment-models-around-world-how-they-work-their-impact

2. Steenhuis S, STRUIJS J, KOOLMAN X, KET J, VAN DER HIJDEN E. Unraveling the Complexity in the Design and Implementation of Bundled Payments: A Scoping Review of Key Elements From a Payer's Perspective. Milbank Q [Internet]. 2020 Mar [cited 2020 Nov 28];98(1):197–222. Available from: https://pubmed.ncbi.nlm.nih.gov/31909852/

3. Ridgely MS, de Vries D, Bozic KJ, Hussey PS. Bundled payment fails to gain a foothold in california: The experience of the IHA bundled payment demonstration. Health Aff [Internet]. 2014 Aug 2 [cited 2021 Mar 24];33(8):1345–52. Available from: https://pubmed.ncbi.nlm.nih.gov/25092835/

4. Hussey PS, Susan Ridgely M, Rosenthal MB. The PROMETHEUS bundled payment experiment: Slow start shows problems in implementing new payment models. Health Aff. 2011 Nov 2;30(11):2116–24.

5. Busse R, Stahl J. Integrated Care Experiences And Outcomes In Germany, The Netherlands, And England. Health Aff [Internet]. 2014 Sep 2 [cited 2021 Mar 10];33(9):1549–58. Available from: http://www.healthaffairs.org/doi/10.1377/hlthaff.2014.0419

6. Stokes J, Struckmann V, Kristensen SR, Fuchs S, van Ginneken E, Tsiachristas A, et al. Towards incentivising integration: A typology of payments for integrated care. Vol. 122, Health Policy. Elsevier Ireland Ltd; 2018. p. 963–9.

7. Tummers JFMM, Schrijvers AJP, Visser-Meily JMA. A qualitative study of stakeholder views on the effects of provider payment on cooperation, quality of care and cost-containment in integrated stroke care. BMC Health Serv Res [Internet]. 2013 Dec 4 [cited 2021 Mar 10];13(1):127. Available from: http://bmchealthservres.biomedcentral.com/articles/10.1186/1472-6963-13-127

8. Conrad DA, Vaughn M, Grembowski D, Marcus-Smith M. Implementing Value-Based Payment Reform. Med Care Res Rev [Internet]. 2016 Aug 1 [cited 2021 Mar 10];73(4):437–57. Available from: http://journals.sagepub.com/doi/10.1177/1077558715615774

9. Matchar DB, Nguyen H V., Tian Y. Bundled Payment and Care of Acute Stroke: What Does it Take to Make it Work? Stroke [Internet]. 2015 May 1 [cited 2018 Nov 20];46(5):1414–21. Available from: http://stroke.ahajournals.org/cgi/doi/10.1161/STROKEAHA.115.009089

10. de Vries EF, Drewes HW, Struijs JN, Heijink R, Baan CA. Barriers to payment reform: Experiences from nine Dutch population health management sites. Health Policy (New York). 2019 Nov 1;123(11):1100–7.

11. Kondo KK, Damberg CL, Mendelson A, Motu'apuaka M, Freeman M, O'Neil M, et al. Implementation Processes and Pay for Performance in Healthcare: A Systematic Review. J Gen Intern Med [Internet]. 2016 Apr 7 [cited 2021 May 12];31(S1):61–9. Available from: http://link.springer.com/10.1007/s11606-015-3567-0

12. Conrad DA. The Theory of Value-Based Payment Incentives and Their Application to Health Care. Health Serv Res [Internet]. 2015 Dec 1 [cited 2021 Jun 4];50:2057–89. Available from: https://onlinelibrary.wiley.com/doi/full/10.1111/1475-6773.12408

13. Voogd E. BARRIERS AND FACILITATORS TO IMPLEMENTING SHARED SAVINGS MODELS IN THE

DUTCH HEALTHCARE SYSTEM A healthcare provider perspective. 2020;46.

14. Gustafson DH, Sainfort F, Eichler M, Adams L, Bisognano M, Steudel H. Developing and testing a model to predict outcomes of organizational change. Health Serv Res [Internet]. 2003 Apr [cited 2021 Jun 15];38(2):751–76. Available from: /pmc/articles/PMC1360903/
