# Supplementary material for: Factors Influencing the Introduction of Value-Based Payment in Integrated Stroke Care: Evidence from a Qualitative Case Study
Source: Int J Integr Care. 2023 Aug 10;23(3):7. doi: 10.5334/ijic.7566 (PMC10437137; doi:10.5334/ijic.7566)
Supplement: Appendix 2. — Integrated care case description. [file ijic-23-3-7566-s2.pdf]

Appendix 2 - Description of the PAVIAS program

To facilitate better care delivery and collaboration among providers and add value for ischemic stroke patients, a BP contract with financial-risk sharing was agreed upon between Erasmus MC and three rehabilitation care providers (Rijndam Rehabilitation, Transmitt Rehabilitation, and Laurens). In addition, a major health insurer (Zilveren Kruis, ZK) was involved as a contract partner and purchaser. Although all stroke patients treated by the providers might benefit from this program and receive the same standard of care regardless of the insurer at which they are enrolled, the contract only pertains to care provided to patients insured with ZK. The PAVIAS program was conceptualized and designed during the period 2017-2018 and came into effect on January 1, 2019. The aim was to change the financial incentives through a shared savings/losses arrangement, thereby creating a shared responsibility to optimize outcomes and contain costs of the integrated stroke care service. The program explicitly aimed to realize the routine measurement and reporting of pre-defined patient-relevant outcomes, both clinical and patient-reported. The overall goal of PAVIAS is to enable value-driven healthcare to ischemic stroke patients by a network of different providers in the region.

Patients admitted to Erasmus MC due to ischemic stroke or transient ischemic attack are included in the program, except patients who are referred by other hospitals or who are below the age of 18 years. Data are collected for all ischemic stroke patients, regardless of where patients are insured. All care related to stroke provided by the abovementioned providers is included in the bundle. Primary care was excluded at this stage to reduce complexity and enhance feasibility.

For the first year (2019), providers agreed to a ‘pay-for-reporting’ structure in which providers would only share in savings relative to a predefined spending benchmark, if they demonstrate ability to report quality/outcome metrics related to patient experience (using a standardize questionnaire, Picker Institute, UK), quality of life (using the EQ-5D-5L), and degree of patient disability (using the modified Rankin Scale [mRS]). From 2020 onwards, providers also bear risk for losses and are required to achieve pre-defined performance targets for the above metrics to qualify for shared savings. Any shared savings/losses will be proportionally distributed among the involved providers based on a predefined distribution key (i.e., EMC 69%, Rijndam 16%, Laurens 14%, Transmitt 1%) and the insurer.
