# Supplementary material for: Factors Influencing the Introduction of Value-Based Payment in Integrated Stroke Care: Evidence from a Qualitative Case Study
Source: Int J Integr Care. 2023 Aug 10;23(3):7. doi: 10.5334/ijic.7566 (PMC10437137; doi:10.5334/ijic.7566)
Supplement: Appendix 3. — Interview guide. [file ijic-23-3-7566-s3.pdf]

Appendix 3. Interview guide

Topics	Questions
**Introduction & consent**	• Brief explanation to respondent about research and purpose (insight into factors/mechanisms of introduction (V)BP) for this interview.<br>• Have you read, understood, and signed the 'informed consent form' and/or are there any remaining questions about this?<br>• Would you please introduce yourself and tell us about your current position(s)?
**Organizational role/ realisation of program**	• Could you please give a description of the program in your own words?<br>• What is or has been your role regarding this program?<br>• And more broadly, what is/was the role of your organisation?<br>• When applicable, what has changed or needs to change in your organization to make this program possible?<br>• What were the reasons for making the above adjustments in your organization?
**Program goals / Outcomes (O)**	• Can you tell me what goals you (/your organization) have/has with the inception of this program?<br>• Is there a hierarchy in these goals?<br>• Do you think these goals align over stakeholders? Why yes or no?
**Contextual factors/ Related mechanisms**	• What factors most contributed to the introduction of this program? How, why, and in what way?<br>• What factors were perceived as barriers to the introduction of this program in your opinion? How, why and in what way?<br>• When applicable, were these barriers addressed? If yes, how and to what extend?
**Exploration of Mechanisms identified in literature review (figure 1)**	• What role did legislation (e.g., financing, privacy) play during the introduction of the program? Did this influence perceived control over the program? In what way and/or how were issues addressed? Can you name examples?<br>• In what way was distribution of financial risk bearing discussed and agreed upon? Do you have examples? Was there any reluctance? If so, how was this addressed and to what extend?<br>• What was the role of trust among stakeholders during the introduction of the program? In what way did this impact introduction? Can you name examples?<br>• What was the role of existing evidence on the effects of (V)BP? In what way did this impact motivation? Was this addressed in any way?<br>• What was the role of information technology? Were these systems sufficient, why yes/no? can you name examples?<br>• How was possible financial risk caused by populations of more comorbid patients addressed? What role did feasibility play in this discussion? Do you have any examples?<br>• How were medical and financial responsibility demarcated between providers? Was there any (individual/organizational) confusion on this? Do you have any examples?

Topics	Questions
	• How do you value (more) intensive collaboration between stakeholders? What role did historic working relations play in this? Did this influence control over the program and if so, how?<br>• How were outcomes and costs defined over stakeholders? How did this process go? Was there a shared desire for data uniformity? If so, what drove this desire? Do you have examples?<br>• How do you rate the effort made by different stakeholders? Was this balanced? Is the potential reward worth the (extra) effort in your opinion? Why yes/no?<br>• How do you rate the possibly of data manipulation (for financial profit)? What role did shared goals and motivation have to that regard? Do you have any examples?<br>• How do you value freedom of choice for patients who might want care providers outside the bundle? Would this be a factor of consideration? Why yes/no?<br>• To what extend did knowledge/experience on (V)BP in the motivation to contribute? Was this addressed in any way? Why yes/no?<br>• To what extend did you perceive support from management? Do you have examples and/or what did you miss? Was this addressed?
**Key factors, mechanism, and lessons**	• (V)PB programs often fail prior to getting introduced. What key factors made the introduction of this program happen, and what was the role of organization in this?<br>• What are the key lessons you have learned during the introduction of this program?<br>• Reflecting on the introduction, what do you think should have been done differently? By you, your organisation, and other stakeholders?<br>• To what extend are you/your organization interested in new or additional (V)BP programs considering your experiences?<br>• Is there anything else we should know to better understand mechanisms related to the introduction of this program?
**Closing remarks**	• Do you have any questions or remarks following this interview?<br>• This interview will be transcribed. I would like to present the preliminary results of this to you to check for factual inaccuracies. Are you willing to do that?
